# An examination of causal associations and shared risk factors for diabetes and cardiovascular diseases in the East Asian population: A Mendelian randomization study

**DOI:** 10.3389/fendo.2023.1132298

**Published:** 2023-02-24

**Authors:** Yulin Guo, Jie Gao, Yan Liu, Yanxiong Jia, Xiangguang An, Xitao Zhang, Pixiong Su

**Affiliations:** Department of Cardiac Surgery, Heart Center and Beijing Key Laboratory of Hypertension, Beijing Chaoyang Hospital, Capital Medical University, Beijing, China

**Keywords:** diabetes, cardiovascular diseases, coronary artery disease, atrial fibrillation, Mendelian randomization

## Abstract

**Background:**

One of the major contributors to disability and mortality among diabetics is cardiovascular disease (CVD), with coronary artery disease (CAD) as the most prevalent type. However, previous studies have provided controversial evidence linking diabetes to other types of CVDs, such as atrial fibrillation (AF). In addition, the risk factors that predispose people to the risk of diabetes and its complications differ across ethnicities, but the disease risk profiles in the East Asian population have been less investigated.

**Methods:**

The causal association between type 2 diabetes (T2D) and two types of CVDs (i.e., AF and CAD) in the East Asian population was first studied using Mendelian randomization (MR) analyses. Next, we examined the causal effect of 49 traits on T2D and CAD to identify their separate and shared risk factors in East Asians. A causal mediation analysis was performed to examine the role of T2D in mediating the relationship between the identified shared risk factors and CAD.

**Results:**

T2D was causally associated with CAD, but not AF, in East Asians. A screening of the risk factors indicated that six and 11 traits were causally associated with T2D and CAD, respectively, with suggestive levels of evidence. Alkaline phosphatase (ALP) was the only trait associated with both T2D and CAD, as revealed by the univariable MR analyses. Moreover, the causal association between ALP and CAD no longer existed after adjusting T2D as a covariable in the causal mediation study.

**Conclusion:**

Our study highlights the risk profiles in the East Asian population, which is important in formulating targeted therapies for T2D and CVDs in East Asians.

## Introduction

Up to 8.8% of the world’s population suffers from diabetes, and International Diabetes Federation projections reveal that by 2040, the number of incidences will have risen to 642 million (1). One of the main contributors to disability among patients with diabetes is cardiovascular disease (CVD) (2, 3). The percentage of people with CVD is higher in diabetic patients than in adults without diabetes (4). CVD leads to the death of roughly 70% of type 2 diabetic patients at and above 65 years old (5). To elucidate, a systematic review that included 4,549,481 type 2 diabetes (T2D) patients showed an overall CVD prevalence of 32.2% (2). Coronary artery disease (CAD) (21.2%) was the most common kind of CVD reported (2). However, previous works have led to controversial conclusion about the association between diabetes and a particular type of CVD, such as atrial fibrillation (AF), the most prevalent type of arrhythmia in the world (6). For example, a study using a cohort of patients having new-onset AF did not establish the association between the symptoms of AF and diabetes (7).

Ethnic disparities in health conditions are well-recognized (8). For example, Asian Indians in the US are more likely to have diabetes, although they have lower chance to be obese (9). In addition, East Asians have more body fat and prone to visceral adiposity at a given body mass index (BMI), which promote the development of diabetes (10). The risk factors that contribute to the development of diabetes complications also differ across Asian and European populations (11). Thus, it is important to understand ethnic differences in disease risk profiles to formulate better treatment strategies.

Mendelian randomization (MR) is a method for inferring causation, which reduces the bias owing to reverse causality and residual confounding. In MR analyses, the genetic instruments are used as a proxy for exposures (12). In causal mediation analyses using a two-step MR design, the direct and indirect effects of exposure on the outcome can also be evaluated (13). Individual-level data was not applied in MR analyses because these analyses use summary statistics from genome-wide association studies (GWAS), which are normally produced using populations with large sample sizes (14). In addition, the availability of GWAS datasets makes it easier to screen disease risk factors at the phenome-wide level (15).

In the current study, we first investigated the potential causal association between T2D and two types of CVDs (i.e., AF and CAD) in the East Asian population. Next, we tested the causal effect of 49 traits on T2D and CAD to identify their separate and shared risk factors in East Asians. A causal mediation analysis was also performed to examine the role of T2D in mediating the relationship between identified shared risk factors and CAD.

## Methods

The GWAS dataset for T2D was obtained from the Diabetes Meta-analysis of Trans-ethnic Association Studies (DIAMANTE) Consortium (16), in which GWAS was performed for the East Asian population. For other traits, the method for traits selection (**Supplementary Figure 1**) was similar to the one used in a recent paper (13). We only included the GWAS summary statistics datasets generated in the Biobank Japan study (17) to ensure that the MR analyses were conducted using genetic data from East Asians. Detailed information was included in **Supplementary Table 1**. The causal relationships between 49 traits (**Supplementary Figure 1**) and T2D/CAD were investigated by univariable MR analyses. For the identified trait (shared risk factor) that can lead to both T2D and CAD, we performed causal mediation analyses, where T2D was deemed as a potential mediator. A reciprocal link between mediator and exposure was not permitted in the mediation studies, so it was necessary to conduct a reverse univariable MR to infer whether these traits could be induced by T2D. The direct effect of trait (shared risk factor) on CAD was estimated using multivariable MR, in which T2D was adjusted as a covariable. The product of the beta coefficient of the effect of trait (shared risk factor) on T2D and the beta coefficient of the association between T2D and CAD (with trait adjusted as covariable) represented the indirect effects of trait (shared risk factor) on CAD.

In the univariable MR studies, the instrumental variables (IVs) used for exposure traits were selected according to various factors. First, the phenotypes should be highly associated with IVs (*P* < 5×10^−8^). Second, a linkage disequilibrium (LD) of R^2^ < 0.001 and clumping with a 10-Mb window were used to ensure that the IVs were not related to each other. Third, each trait’s IVs should have at least five variants as biallelic single-nucleotide polymorphisms (SNPs). In the univariable MR studies, the inverse-variance weighted (IVW) method, weighted median method, and MR-Egger were used, with the IVW approach being regarded as the primary method. Potential horizontal pleiotropy was examined using the MR-Egger intercept test. A 5% false-discovery rate (FDR) was used to correct multiple comparisons. The code for the MR studies was modified from a recent work (13), in which the R packages TwoSampleMR and MVMR, respectively, were applied to conducted the MR analyses.

## Results

**Figure 1:**
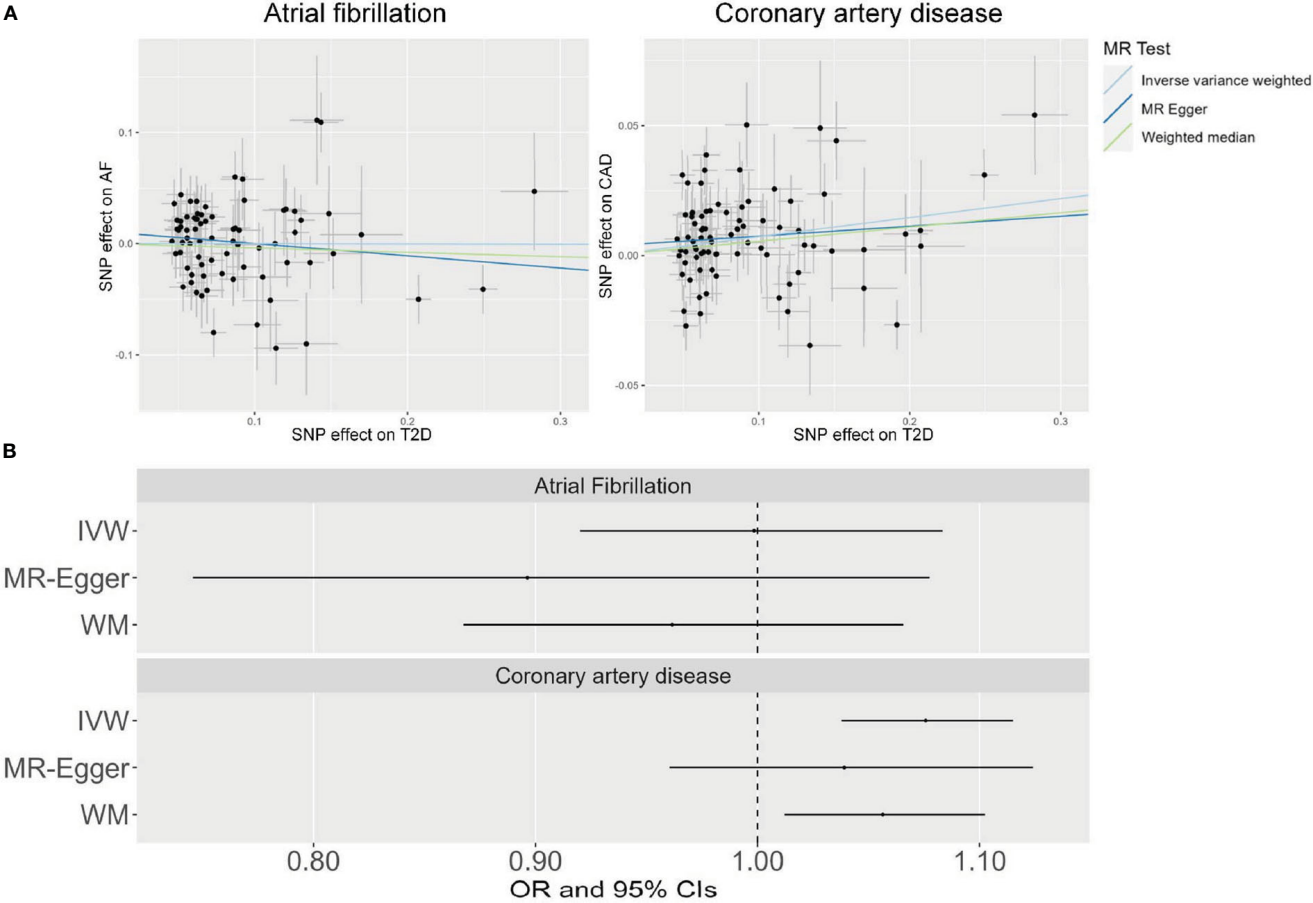
Scatter plots **(A)** and forest plots **(B)** showing the results of Mendelian randomization (MR) analyses studying the causal association between T2D and cardiovascular diseases in the East Asian population.

The results of the MR analysis using the IVW approach indicated a significant association between genetically predicted T2D and CAD (*P* = 6.63×10^−5^) (**Figure 1** and **Supplementary Figure 2**). However, no causal association between T2D and AF was observed (*P* = 0.97) (**Figure 1** and **Supplementary Figure 2**). The same relationship trajectory was apparent in the MR sensitivity analyses using the weighted median and MR-Egger methods (**Figure 1**). Moreover, a leave-one-out sensitivity analysis suggested that not a single SNP was responsible for the causal effect of T2D on CAD (**Supplementary Figure 3**). The intercept term of the MR-Egger method was applied to examine the horizontal pleiotropy, which revealed that it was not significant (*P* = 0.34) in the studies.

**Figure 2:**
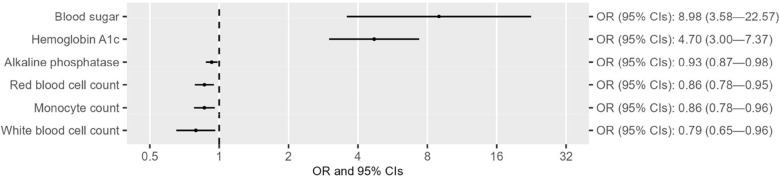
Forest plots showing the causal effect of traits on T2D with suggestive levels of evidence (*P* < 0.05) in the East Asian population.

**Figure 3:**
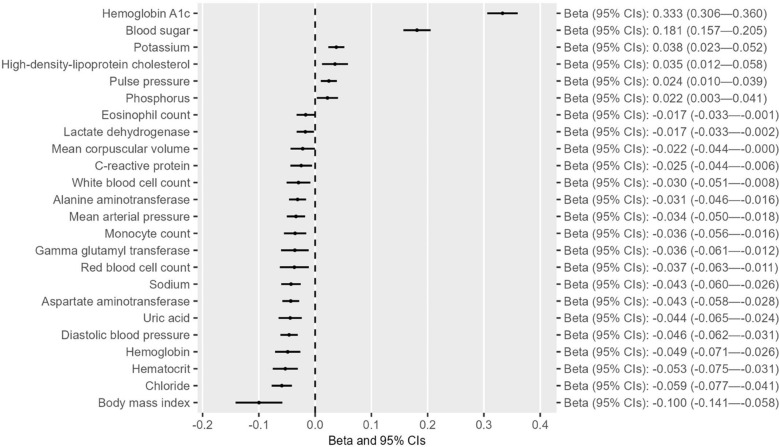
Forest plots showing the causal effect of T2D on traits with suggestive levels of evidence (*P* < 0.05) in the East Asian population.

**Figure 4:**
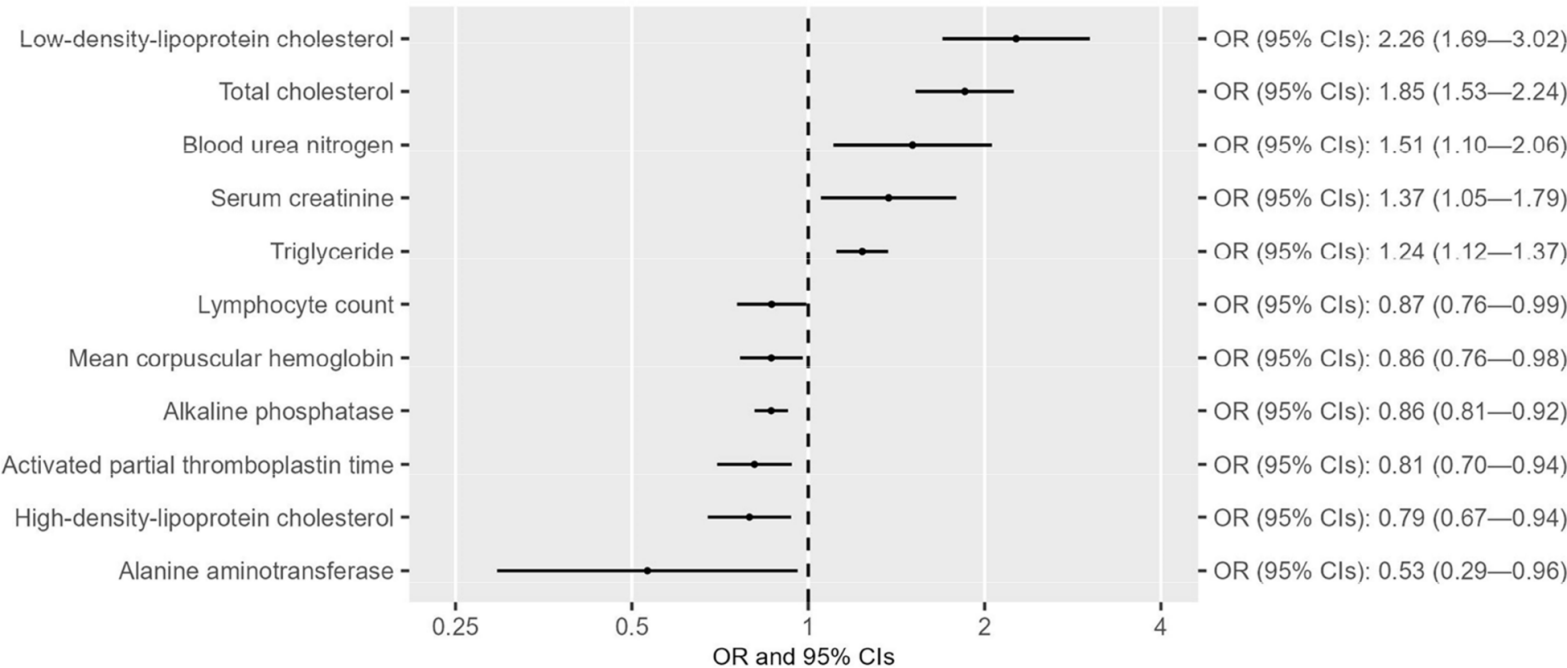
Forest plots showing the causal effect of traits on CAD with suggestive levels of evidence (*P* < 0.05) in the East Asian population.

**Figure 5:**
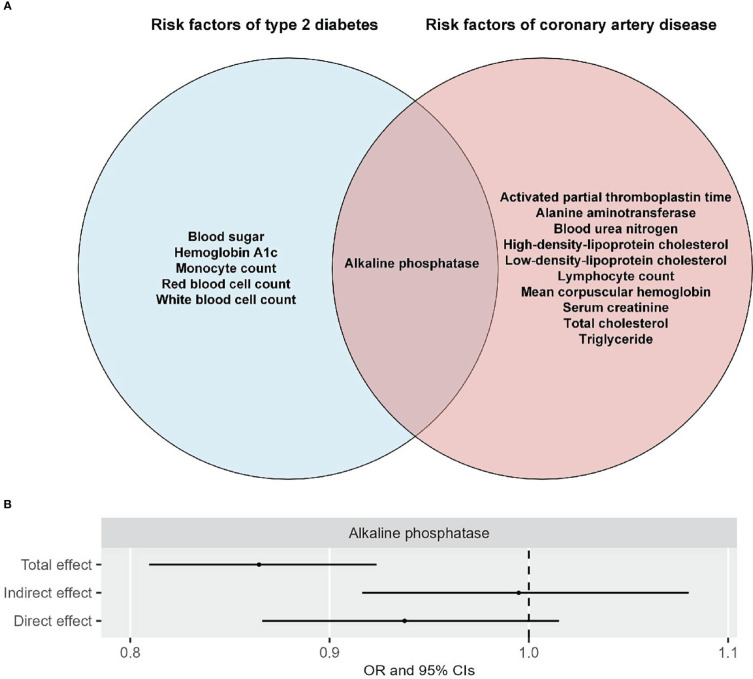
Shared and independent risks of T2D and CAD are presented in a Venn diagram **(A)**, and the total, indirect, and direct effects of alkaline phosphatase (ALP) on CAD are studied by causal mediation analyses **(B)** in the East Asian population.

After confirming the causal effect of T2D on CAD in the East Asian population, we next examine the separate and shared risk factors of these two diseases by including the GWAS summary datasets of 49 traits from the Biobank Japan study (**Supplementary Table 1**), in accordance with the criteria indicated in the flowchart (**Supplementary Figure 1**). Univariable MR analyses indicated that out of the 49 traits, six were associated with T2D at suggestive levels of evidence (*P* < 0.05) (**Figure 2** and **Supplementary Tables 2**-**3**). Three of these six traits (i.e., hemoglobin A1c, blood sugar, and red blood cell count) survived 5% FDR correction for multiple comparisons (**Supplementary Table 4**). Reverse MR analyses suggested that alkaline phosphatase was the only trait that could not be altered by T2D (**Figure 3** and **Supplementary Tables 2**-**3**). Eleven of 49 traits showed causal association with CAD at suggestive levels of evidence (*P* < 0.05) (**Figure 4** and **Supplementary Tables 2**-**3**), and six of the 11 traits, namely, total cholesterol (TC), triglycerides, low-density-lipoprotein cholesterol (LDL), high-density lipoprotein cholesterol (HDL), alkaline phosphatase (ALP), and activated partial thromboplastin time (APTT), survived 5% FDR correction (**Supplementary Table 4**). Thus, the results revealed that ALP was causally associated with both T2D and CAD (**Figure 5A**), and the following causal mediation analysis based on two-step MR indicated that ALP was no longer associated with CAD after adjusting T2D as a covariable in the multivariable MR (**Figure 5B**).

## Discussion

In the present study, the results of MR analyses suggested that T2D was causally associated with CAD, but not AF, in East Asians. The screening of the risk factors indicated that six and 11 traits were causally associated with T2D and CAD, respectively, with suggestive levels of evidence. ALP was the only trait associated with both T2D and CAD, as revealed by the univariable MR analyses. The causal association between ALP and CAD no longer existed after adjusting T2D as a covariable in the causal mediation study (direct effect).

T2D can approximately shorten life expectancy by a decade, and CVD is a major cause of death in T2D patients (18). However, the association of T2D and AF, as well as the exact pathophysiology of AF in diabetes patients, has not been fully established (19). The Framingham Heart Study correlated elevated glycemic levels with an increased risk of AF (20). Moreover, diabetes patients with AF had increased rates of overall and cardiovascular mortality, coupled with a decline in life quality compared with patients who only had AF but were not diabetic (21). However, a correlation between diabetes and non-paroxysmal AF was not observed (22). Diabetes cannot independently lead to AF after confounder adjustment, according to a survey in China (23). Thus, it is still unknown whether there is a causative association between diabetes and AF. Our MR study using genetic data from the East Asian population suggested that diabetes could not causally lead to AF.

CVD is a significant contributor to comorbidity and mortality among T2D patients, with CAD having the highest prevalence rate (2). Research has indicated that patients with diabetes have a higher susceptibility to CAD compared with non-diabetics (24). We consistently observed a causal association of diabetes with CAD in the East Asian population. Several reasons, such as insulin resistance, dyslipidemia, and hyperglycemia, have been postulated to explain the high vulnerability to CAD among patients with diabetes. These processes can be linked to abnormal functioning of the platelets, causing vascular smooth muscle dysfunction, and irregularity in the functioning of endothelial cells (25). Indeed, atherosclerotic plaques in diabetic patients are often lipid-laden, making them more prone to rupture compared with those of people without diabetes (26). In addition, critical to atherosclerosis is the process of inflammation, whose activation in T2D is often linked to insulin resistance and obesity (27). Hyperglycemia has also been linked with the promotion of epigenetic alterations that initiate the over-expression of genes linked to vascular inflammation, thus establish a basis for atherosclerosis and endothelial dysfunction (28).

The Collaborative Analysis of Diagnostic criteria in Europe (DECODE) study indicated that the prevalence of diabetes was higher in urban Chinese and Japanese patients aged 30–69 years than in Europeans (29). Young patients have a higher chance to experience β-cell failure and long-lasting disease, making them have a higher risk for microvascular and macrovascular problems (10). For example, patients with T2D from East Asia are more likely than those from Europe to experience renal issues (10). One of the potential reasons for this interethnic disparity is that Asians, at a given BMI, usually have higher visceral adiposity compared to Caucasians, which is likely to be more harmful and can cause insulin resistance (30). For example, American Japanese patients have higher level of visceral adiposity than their Caucasian counterparts (31). For other race, the association between metabolic parameters and CVD can be different in Black and White population, and ethnicity is also responsible for the disparities in the metabolic syndrome associated CVD and T2D (32). Because of the ethnic differences in risk profiles, we screened and identified the independent and shared risk factors of diabetes and CAD using GWAS summary data generated from the East Asian population.

Red blood cell (RBC) changes are likely to happen in diabetes patients (33). For example, red blood cell parameters are correlated with glycemic control among adult patients with T2D in Eastern Ethiopia (34). Consistent with the literature, the causality interference by MR analyses in the present study suggested that RBC count was negatively associated with diabetes risk. Long-term hyperglycemia leads to the production of free oxygen radicals and the irreversible glycation of hemoglobin and RBC membrane proteins, resulting in a relative drop in RBC count (35). Thus, these processes make RBCs become less deformable and have a reduced chance of survival (36).

A moderate to very significant correlation between triglyceride levels and the risk of coronary heart disease has been observed (37). The measurement of TC is helpful in estimating CVD risk and making clinical decision for the start of statin therapy (38). Indeed, the risk of coronary heart disease increases by 24% for males and 20% for females for every 1 mmol/L increase in TC (39). For LDL cholesterol, angiographic trials confirm the significance of LDL cholesterol reduction in reducing the risk of CAD (40). Widespread epidemiological research indicates that low levels of HDL are a sign of increased cardiovascular risk (41). Consistent with these clinical observations, our MR analyses identified the causal association between lipid profile and CAD in the East Asian population. As a common coagulation screening test, the measurement of APTT can be used to estimate intrinsic coagulation pathway activity (42). The degree and severity of coronary stenosis can be estimated by using APTT in individuals undergoing coronary angiography; notably, the patients who had ST-Segment Elevation Myocardial Infarction (STEMI) had low APTT values (43). Moreover, a short APTT is correlated with higher thrombin production and an increased risk for thrombosis (44). Consistently, our study revealed that higher APTT was causally associated with decreased CAD risk in the East Asian population.

ALP is a plasma membrane-anchored enzyme that is widely present in nature (45). A correlation between baseline serum ALP levels and new-onset diabetes has been established in hypertensive individuals (46). In an Iranian population, the level of ALP and the risk of coronary heart disease were independently correlated (47). However, ALP was not associated with diabetes, according to the results of MR research, which only included persons of European ancestry (48). Our MR analysis in an East Asian population indicated that ALP was negatively associated with both diabetes and CAD, and the association between ALP and CAD was not significant after adjusting T2D in the multivariable MR. Mechanistically, ALP reduce the bioavailability of nitric oxide (NO), leading to an altered endothelial NO synthase activity (46). Besides serum ALP, intestinal alkaline phosphatase (IAP), as a membrane-bound glycoprotein mainly expressed in proximal small intestine, is also related to T2D (49). For instance, T2D can be observed in mice lacking IAP (50). Additionally, oral administration of IAP protects and even reverses high-fat-diet-induced T2D in wild-type mice by reducing metabolic endotoxemia and detoxifying lipopolysaccharides (LPS) (49).

This study has several limitations. First, a relatively high level of multiple comparison burden may exist when many traits are included in the analyses. To address this point, we also presented the results with suggestive levels of evidence. Second, as an inherent drawback, an MR study cannot completely rule out the potential horizontal pleiotropy. Thus, we used multiple MR methods as sensitivity analyses to enhance the credibility of our conclusion.

## Conclusion

T2D is causally associated with CAD, but not AF, in the East Asian population. Multiple traits were identified as separate risk factors of T2D or CAD. A mediating effect of T2D on the association between ALP and CAD was observed. Our study highlights the risk profiles in the East Asian population, which is important for formulating targeted therapies for T2D and CVDs in East Asians.

## Data availability statement

The original contributions presented in the study are included in the article/**Supplementary Material**. Further inquiries can be directed to the corresponding authors.

## Author contributions

YG and PS: conception and design, data analysis, and interpretation. JG, YL, YJ, XA, and XZ: collection and assembly of data, and prepared the manuscript. All authors contributed to the article and approved the submitted version.
