# Supplementary material for: An examination of causal associations and shared risk factors for diabetes and cardiovascular diseases in the East Asian population: A Mendelian randomization study
Source: Front Endocrinol (Lausanne). 2023 Feb 24;14:1132298. doi: 10.3389/fendo.2023.1132298 (PMC9999111; doi:10.3389/fendo.2023.1132298)
Supplement: Supplementary file 1 [file DataSheet_1.docx]

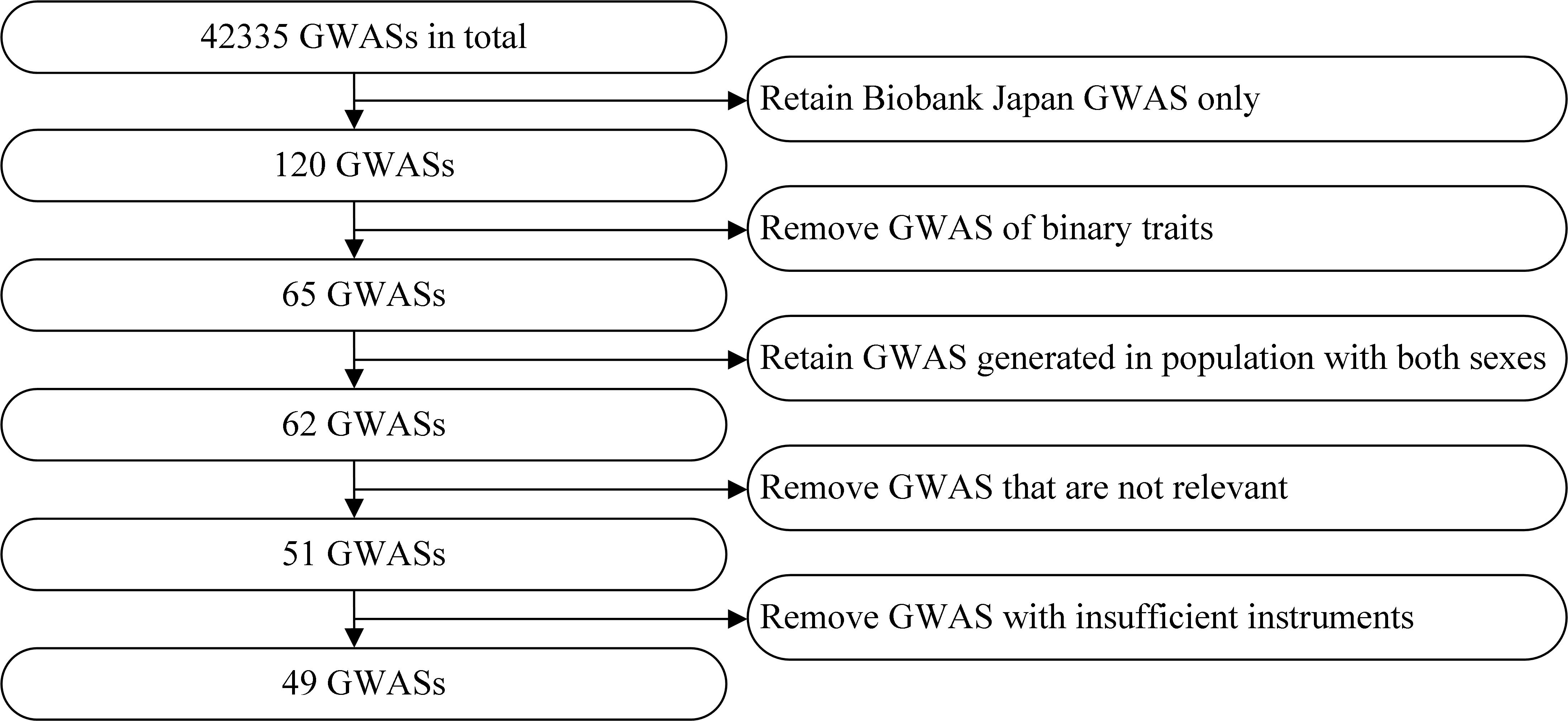


Supplementary Figure 1. The procedure applied in the trait selection





Supplementary Figure 2. Funnel plot of Mendelian randomization (MR) analyses indicating the causal association between T2D and cardiovascular diseases in the East Asian population


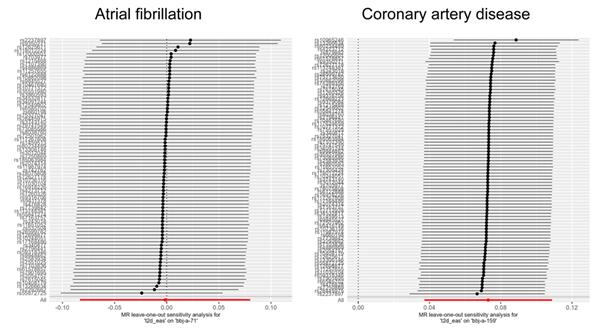


Supplementary Figure 3. Plots of leave-one-out analysis for the causal effect of T2D on cardiovascular diseases

Supplementary Table 1. GWAS summary datasets for the traits

| Trait | ID | Sex | Population | Sample size |
| --- | --- | --- | --- | --- |
| Activated partial thromboplastin time | bbj-a-7 | Males and Females | East Asian | 37767 |
| Alanine aminotransferase | bbj-a-6 | Males and Females | East Asian | 134182 |
| Albumin | bbj-a-9 | Males and Females | East Asian | 102223 |
| Albumin/globulin ratio | bbj-a-4 | Males and Females | East Asian | 98626 |
| Alkaline phosphatase | bbj-a-5 | Males and Females | East Asian | 105030 |
| Aspartate aminotransferase | bbj-a-8 | Males and Females | East Asian | 134154 |
| Basophil count | bbj-a-12 | Males and Females | East Asian | 62076 |
| Blood sugar | bbj-a-10 | Males and Females | East Asian | 93146 |
| Blood urea nitrogen | bbj-a-11 | Males and Females | East Asian | 139818 |
| Body mass index | bbj-a-1 | Males and Females | East Asian | 158284 |
| Calcium | bbj-a-15 | Males and Females | East Asian | 71701 |
| Chloride | bbj-a-16 | Males and Females | East Asian | 126402 |
| C-reactive protein | bbj-a-14 | Males and Females | East Asian | 75391 |
| Creatine kinase | bbj-a-13 | Males and Females | East Asian | 106080 |
| Diastolic blood pressure | bbj-a-17 | Males and Females | East Asian | 136615 |
| Eosinophil count | bbj-a-20 | Males and Females | East Asian | 62076 |
| Estimated glomerular filtration rate | bbj-a-60 | Males and Females | East Asian | 143658 |
| Gamma glutamyl transferase | bbj-a-23 | Males and Females | East Asian | 118309 |
| Height | bbj-a-70 | Males and Females | East Asian | 159095 |
| Hematocrit | bbj-a-27 | Males and Females | East Asian | 108757 |
| Hemoglobin | bbj-a-25 | Males and Females | East Asian | 108769 |
| Hemoglobin A1c | bbj-a-26 | Males and Females | East Asian | 42790 |
| High-density-lipoprotein cholesterol | bbj-a-24 | Males and Females | East Asian | 70657 |
| Lactate dehydrogenase | bbj-a-30 | Males and Females | East Asian | 126319 |
| Low-density-lipoprotein cholesterol | bbj-a-31 | Males and Females | East Asian | 72866 |
| Lymphocyte count | bbj-a-36 | Males and Females | East Asian | 62076 |
| Mean arterial pressure | bbj-a-37 | Males and Females | East Asian | 136482 |
| Mean corpuscular hemoglobin | bbj-a-38 | Males and Females | East Asian | 108054 |
| Mean corpuscular volume | bbj-a-40 | Males and Females | East Asian | 108256 |
| Menarche | bbj-a-62 | Males and Females | East Asian | 67029 |
| Menopause | bbj-a-63 | Males and Females | East Asian | 43861 |
| Monocyte count | bbj-a-41 | Males and Females | East Asian | 62076 |
| Neutrophil count | bbj-a-44 | Males and Females | East Asian | 62076 |
| Non-albumin protein | bbj-a-42 | Males and Females | East Asian | 98538 |
| Phosphorus | bbj-a-45 | Males and Females | East Asian | 42793 |
| Platelet count | bbj-a-49 | Males and Females | East Asian | 108208 |
| Potassium | bbj-a-29 | Males and Females | East Asian | 132938 |
| Prothrombin time | bbj-a-47 | Males and Females | East Asian | 58110 |
| Pulse pressure | bbj-a-46 | Males and Females | East Asian | 136249 |
| Red blood cell count | bbj-a-50 | Males and Females | East Asian | 108794 |
| Serum creatinine | bbj-a-61 | Males and Females | East Asian | 142097 |
| Sodium | bbj-a-43 | Males and Females | East Asian | 127304 |
| Systolic blood pressure | bbj-a-52 | Males and Females | East Asian | 136597 |
| Total bilirubin | bbj-a-53 | Males and Females | East Asian | 110207 |
| Total cholesterol | bbj-a-54 | Males and Females | East Asian | 128305 |
| Total protein | bbj-a-56 | Males and Females | East Asian | 113509 |
| Triglyceride | bbj-a-55 | Males and Females | East Asian | 105597 |
| Uric acid | bbj-a-57 | Males and Females | East Asian | 109029 |
| White blood cell count | bbj-a-58 | Males and Females | East Asian | 107964 |

Supplementary Table 2. MR analyses indicating the effect of traits on T2D/CAD and the effect of T2D on traits

| Exposure | Outcome | Method | Nsnp | Beta | SE | P |
| --- | --- | --- | --- | --- | --- | --- |
| Activated partial thromboplastin time | T2D | IVW | 13 | -0.078 | 0.046 | 8.67E-02 |
| Activated partial thromboplastin time | T2D | MR Egger | 13 | 0.035 | 0.079 | 6.65E-01 |
| Activated partial thromboplastin time | T2D | WM | 13 | -0.005 | 0.029 | 8.55E-01 |
| Alanine aminotransferase | T2D | IVW | 21 | 0.110 | 0.147 | 4.56E-01 |
| Alanine aminotransferase | T2D | MR Egger | 21 | 0.495 | 0.505 | 3.40E-01 |
| Alanine aminotransferase | T2D | WM | 21 | -0.015 | 0.116 | 8.98E-01 |
| Albumin | T2D | IVW | 15 | -0.210 | 0.125 | 9.36E-02 |
| Albumin | T2D | MR Egger | 15 | -0.650 | 0.334 | 7.32E-02 |
| Albumin | T2D | WM | 15 | -0.113 | 0.099 | 2.55E-01 |
| Albumin/globulin ratio | T2D | IVW | 39 | 0.052 | 0.058 | 3.70E-01 |
| Albumin/globulin ratio | T2D | MR Egger | 39 | 0.160 | 0.124 | 2.05E-01 |
| Albumin/globulin ratio | T2D | WM | 39 | 0.080 | 0.059 | 1.72E-01 |
| Alkaline phosphatase | T2D | IVW | 44 | -0.076 | 0.030 | 1.13E-02 |
| Alkaline phosphatase | T2D | MR Egger | 44 | -0.109 | 0.043 | 1.43E-02 |
| Alkaline phosphatase | T2D | WM | 44 | -0.136 | 0.026 | 2.44E-07 |
| Aspartate aminotransferase | T2D | IVW | 21 | 0.009 | 0.116 | 9.41E-01 |
| Aspartate aminotransferase | T2D | MR Egger | 21 | 0.405 | 0.351 | 2.63E-01 |
| Aspartate aminotransferase | T2D | WM | 21 | 0.197 | 0.100 | 4.97E-02 |
| Basophil count | T2D | IVW | 22 | -0.014 | 0.052 | 7.81E-01 |
| Basophil count | T2D | MR Egger | 22 | 0.046 | 0.113 | 6.88E-01 |
| Basophil count | T2D | WM | 22 | -0.015 | 0.058 | 7.96E-01 |
| Blood sugar | T2D | IVW | 15 | 2.196 | 0.470 | 2.97E-06 |
| Blood sugar | T2D | MR Egger | 15 | -0.588 | 2.273 | 8.00E-01 |
| Blood sugar | T2D | WM | 15 | 0.898 | 0.160 | 2.06E-08 |
| Blood urea nitrogen | T2D | IVW | 37 | 0.037 | 0.140 | 7.91E-01 |
| Blood urea nitrogen | T2D | MR Egger | 37 | 0.127 | 0.422 | 7.65E-01 |
| Blood urea nitrogen | T2D | WM | 37 | 0.054 | 0.079 | 4.98E-01 |
| Body mass index | T2D | IVW | 61 | -0.157 | 0.297 | 5.96E-01 |
| Body mass index | T2D | MR Egger | 61 | -0.489 | 0.933 | 6.02E-01 |
| Body mass index | T2D | WM | 61 | 0.918 | 0.099 | 1.22E-20 |
| Calcium | T2D | IVW | 14 | 0.055 | 0.118 | 6.38E-01 |
| Calcium | T2D | MR Egger | 14 | 0.697 | 0.441 | 1.40E-01 |
| Calcium | T2D | WM | 14 | 0.200 | 0.100 | 4.44E-02 |
| Chloride | T2D | IVW | 13 | -0.645 | 0.650 | 3.21E-01 |
| Chloride | T2D | MR Egger | 13 | 1.550 | 3.513 | 6.68E-01 |
| Chloride | T2D | WM | 13 | -0.079 | 0.174 | 6.48E-01 |
| C-reactive protein | T2D | IVW | 7 | 0.045 | 0.142 | 7.49E-01 |
| C-reactive protein | T2D | MR Egger | 7 | 0.289 | 0.331 | 4.23E-01 |
| C-reactive protein | T2D | WM | 7 | 0.129 | 0.077 | 9.28E-02 |
| Creatine kinase | T2D | IVW | 32 | -0.032 | 0.092 | 7.27E-01 |
| Creatine kinase | T2D | MR Egger | 32 | -0.618 | 0.229 | 1.14E-02 |
| Creatine kinase | T2D | WM | 32 | -0.097 | 0.072 | 1.79E-01 |
| Diastolic blood pressure | T2D | IVW | 15 | -0.069 | 0.197 | 7.24E-01 |
| Diastolic blood pressure | T2D | MR Egger | 15 | 1.269 | 0.484 | 2.11E-02 |
| Diastolic blood pressure | T2D | WM | 15 | -0.068 | 0.140 | 6.29E-01 |
| Eosinophil count | T2D | IVW | 16 | 0.034 | 0.063 | 5.95E-01 |
| Eosinophil count | T2D | MR Egger | 16 | 0.164 | 0.226 | 4.79E-01 |
| Eosinophil count | T2D | WM | 16 | 0.096 | 0.065 | 1.42E-01 |
| Estimated glomerular filtration rate | T2D | IVW | 53 | 0.013 | 0.093 | 8.87E-01 |
| Estimated glomerular filtration rate | T2D | MR Egger | 53 | 0.241 | 0.301 | 4.27E-01 |
| Estimated glomerular filtration rate | T2D | WM | 53 | -0.032 | 0.074 | 6.66E-01 |
| Gamma glutamyl transferase | T2D | IVW | 56 | 0.059 | 0.046 | 1.96E-01 |
| Gamma glutamyl transferase | T2D | MR Egger | 56 | 0.009 | 0.076 | 9.07E-01 |
| Gamma glutamyl transferase | T2D | WM | 56 | 0.059 | 0.036 | 1.02E-01 |
| Height | T2D | IVW | 412 | 0.000 | 0.002 | 9.52E-01 |
| Height | T2D | MR Egger | 412 | 0.002 | 0.002 | 3.11E-01 |
| Height | T2D | WM | 412 | 0.000 | 0.002 | 8.17E-01 |
| Hematocrit | T2D | IVW | 18 | -0.115 | 0.107 | 2.84E-01 |
| Hematocrit | T2D | MR Egger | 18 | -0.333 | 0.335 | 3.35E-01 |
| Hematocrit | T2D | WM | 18 | -0.126 | 0.107 | 2.41E-01 |
| Hemoglobin | T2D | IVW | 15 | -0.077 | 0.102 | 4.51E-01 |
| Hemoglobin | T2D | MR Egger | 15 | -0.412 | 0.320 | 2.19E-01 |
| Hemoglobin | T2D | WM | 15 | -0.067 | 0.101 | 5.04E-01 |
| Hemoglobin A1c | T2D | IVW | 22 | 1.548 | 0.229 | 1.51E-11 |
| Hemoglobin A1c | T2D | MR Egger | 22 | 2.991 | 0.927 | 4.23E-03 |
| Hemoglobin A1c | T2D | WM | 22 | 0.439 | 0.097 | 5.35E-06 |
| High-density-lipoprotein cholesterol | T2D | IVW | 42 | 0.002 | 0.059 | 9.68E-01 |
| High-density-lipoprotein cholesterol | T2D | MR Egger | 42 | 0.001 | 0.108 | 9.89E-01 |
| High-density-lipoprotein cholesterol | T2D | WM | 42 | -0.008 | 0.034 | 8.17E-01 |
| Lactate dehydrogenase | T2D | IVW | 13 | 0.032 | 0.103 | 7.57E-01 |
| Lactate dehydrogenase | T2D | MR Egger | 13 | -0.108 | 0.132 | 4.30E-01 |
| Lactate dehydrogenase | T2D | WM | 13 | -0.009 | 0.039 | 8.12E-01 |
| Low-density-lipoprotein cholesterol | T2D | IVW | 28 | -0.044 | 0.062 | 4.74E-01 |
| Low-density-lipoprotein cholesterol | T2D | MR Egger | 28 | 0.021 | 0.089 | 8.18E-01 |
| Low-density-lipoprotein cholesterol | T2D | WM | 28 | 0.022 | 0.035 | 5.39E-01 |
| Lymphocyte count | T2D | IVW | 10 | -0.124 | 0.120 | 3.00E-01 |
| Lymphocyte count | T2D | MR Egger | 10 | -0.113 | 0.277 | 6.95E-01 |
| Lymphocyte count | T2D | WM | 10 | -0.123 | 0.092 | 1.79E-01 |
| Mean arterial pressure | T2D | IVW | 25 | -0.106 | 0.138 | 4.44E-01 |
| Mean arterial pressure | T2D | MR Egger | 25 | 0.696 | 0.390 | 8.70E-02 |
| Mean arterial pressure | T2D | WM | 25 | -0.233 | 0.103 | 2.29E-02 |
| Mean corpuscular hemoglobin | T2D | IVW | 87 | 0.023 | 0.031 | 4.57E-01 |
| Mean corpuscular hemoglobin | T2D | MR Egger | 87 | 0.105 | 0.062 | 9.24E-02 |
| Mean corpuscular hemoglobin | T2D | WM | 87 | 0.013 | 0.034 | 6.95E-01 |
| Mean corpuscular volume | T2D | IVW | 85 | 0.018 | 0.030 | 5.37E-01 |
| Mean corpuscular volume | T2D | MR Egger | 85 | 0.147 | 0.058 | 1.39E-02 |
| Mean corpuscular volume | T2D | WM | 85 | 0.013 | 0.035 | 7.15E-01 |
| Menarche | T2D | IVW | 7 | 0.037 | 0.055 | 4.95E-01 |
| Menarche | T2D | MR Egger | 7 | -0.336 | 0.385 | 4.23E-01 |
| Menarche | T2D | WM | 7 | 0.034 | 0.069 | 6.26E-01 |
| Menopause | T2D | IVW | 13 | 0.004 | 0.027 | 8.87E-01 |
| Menopause | T2D | MR Egger | 13 | -0.057 | 0.085 | 5.17E-01 |
| Menopause | T2D | WM | 13 | 0.019 | 0.019 | 3.16E-01 |
| Monocyte count | T2D | IVW | 31 | -0.148 | 0.053 | 5.46E-03 |
| Monocyte count | T2D | MR Egger | 31 | 0.346 | 0.168 | 4.89E-02 |
| Monocyte count | T2D | WM | 31 | -0.051 | 0.054 | 3.40E-01 |
| Neutrophil count | T2D | IVW | 20 | -0.123 | 0.074 | 9.91E-02 |
| Neutrophil count | T2D | MR Egger | 20 | 0.224 | 0.214 | 3.09E-01 |
| Neutrophil count | T2D | WM | 20 | -0.114 | 0.062 | 6.66E-02 |
| Non-albumin protein | T2D | IVW | 47 | -0.055 | 0.044 | 2.08E-01 |
| Non-albumin protein | T2D | MR Egger | 47 | -0.118 | 0.093 | 2.09E-01 |
| Non-albumin protein | T2D | WM | 47 | -0.060 | 0.052 | 2.54E-01 |
| Phosphorus | T2D | IVW | 8 | -0.018 | 0.066 | 7.85E-01 |
| Phosphorus | T2D | MR Egger | 8 | -0.138 | 0.276 | 6.34E-01 |
| Phosphorus | T2D | WM | 8 | -0.069 | 0.077 | 3.69E-01 |
| Platelet count | T2D | IVW | 75 | -0.089 | 0.045 | 5.11E-02 |
| Platelet count | T2D | MR Egger | 75 | -0.123 | 0.114 | 2.86E-01 |
| Platelet count | T2D | WM | 75 | -0.032 | 0.046 | 4.88E-01 |
| Potassium | T2D | IVW | 12 | 0.129 | 0.118 | 2.72E-01 |
| Potassium | T2D | MR Egger | 12 | -0.463 | 0.386 | 2.57E-01 |
| Potassium | T2D | WM | 12 | 0.000 | 0.122 | 9.99E-01 |
| Prothrombin time | T2D | IVW | 11 | 0.005 | 0.086 | 9.55E-01 |
| Prothrombin time | T2D | MR Egger | 11 | -0.102 | 0.116 | 3.99E-01 |
| Prothrombin time | T2D | WM | 11 | -0.037 | 0.043 | 3.97E-01 |
| Pulse pressure | T2D | IVW | 16 | 0.006 | 0.157 | 9.69E-01 |
| Pulse pressure | T2D | MR Egger | 16 | 0.476 | 0.975 | 6.33E-01 |
| Pulse pressure | T2D | WM | 16 | -0.048 | 0.124 | 6.96E-01 |
| Red blood cell count | T2D | IVW | 47 | -0.148 | 0.049 | 2.44E-03 |
| Red blood cell count | T2D | MR Egger | 47 | -0.100 | 0.099 | 3.18E-01 |
| Red blood cell count | T2D | WM | 47 | -0.043 | 0.054 | 4.31E-01 |
| Serum creatinine | T2D | IVW | 52 | -0.017 | 0.085 | 8.39E-01 |
| Serum creatinine | T2D | MR Egger | 52 | -0.370 | 0.286 | 2.02E-01 |
| Serum creatinine | T2D | WM | 52 | 0.016 | 0.078 | 8.39E-01 |
| Sodium | T2D | IVW | 13 | -0.316 | 0.221 | 1.52E-01 |
| Sodium | T2D | MR Egger | 13 | -0.367 | 1.300 | 7.83E-01 |
| Sodium | T2D | WM | 13 | -0.187 | 0.142 | 1.89E-01 |
| Systolic blood pressure | T2D | IVW | 20 | -0.121 | 0.133 | 3.62E-01 |
| Systolic blood pressure | T2D | MR Egger | 20 | 0.679 | 0.441 | 1.41E-01 |
| Systolic blood pressure | T2D | WM | 20 | -0.214 | 0.099 | 3.09E-02 |
| Total bilirubin | T2D | IVW | 14 | -0.008 | 0.035 | 8.17E-01 |
| Total bilirubin | T2D | MR Egger | 14 | -0.007 | 0.049 | 8.93E-01 |
| Total bilirubin | T2D | WM | 14 | 0.000 | 0.024 | 9.86E-01 |
| Total cholesterol | T2D | IVW | 43 | -0.033 | 0.068 | 6.28E-01 |
| Total cholesterol | T2D | MR Egger | 43 | 0.070 | 0.127 | 5.85E-01 |
| Total cholesterol | T2D | WM | 43 | 0.028 | 0.052 | 5.94E-01 |
| Total protein | T2D | IVW | 31 | -0.161 | 0.107 | 1.32E-01 |
| Total protein | T2D | MR Egger | 31 | -0.333 | 0.241 | 1.78E-01 |
| Total protein | T2D | WM | 31 | -0.201 | 0.071 | 4.52E-03 |
| Triglyceride | T2D | IVW | 32 | -0.003 | 0.071 | 9.64E-01 |
| Triglyceride | T2D | MR Egger | 32 | -0.013 | 0.116 | 9.13E-01 |
| Triglyceride | T2D | WM | 32 | 0.077 | 0.039 | 5.12E-02 |
| Uric acid | T2D | IVW | 39 | -0.013 | 0.053 | 7.99E-01 |
| Uric acid | T2D | MR Egger | 39 | 0.017 | 0.090 | 8.50E-01 |
| Uric acid | T2D | WM | 39 | -0.023 | 0.040 | 5.69E-01 |
| White blood cell count | T2D | IVW | 34 | -0.234 | 0.099 | 1.88E-02 |
| White blood cell count | T2D | MR Egger | 34 | 0.272 | 0.271 | 3.22E-01 |
| White blood cell count | T2D | WM | 34 | -0.120 | 0.071 | 9.12E-02 |
| Activated partial thromboplastin time | CAD | IVW | 12 | -0.211 | 0.075 | 4.72E-03 |
| Activated partial thromboplastin time | CAD | MR Egger | 12 | -0.227 | 0.257 | 3.97E-01 |
| Activated partial thromboplastin time | CAD | WM | 12 | -0.159 | 0.064 | 1.23E-02 |
| Alanine aminotransferase | CAD | IVW | 20 | -0.632 | 0.301 | 3.60E-02 |
| Alanine aminotransferase | CAD | MR Egger | 20 | -2.898 | 0.834 | 2.70E-03 |
| Alanine aminotransferase | CAD | WM | 20 | -0.225 | 0.131 | 8.61E-02 |
| Albumin | CAD | IVW | 17 | 0.064 | 0.093 | 4.90E-01 |
| Albumin | CAD | MR Egger | 17 | 0.084 | 0.264 | 7.56E-01 |
| Albumin | CAD | WM | 17 | 0.101 | 0.102 | 3.22E-01 |
| Albumin/globulin ratio | CAD | IVW | 40 | 0.012 | 0.061 | 8.39E-01 |
| Albumin/globulin ratio | CAD | MR Egger | 40 | 0.148 | 0.130 | 2.62E-01 |
| Albumin/globulin ratio | CAD | WM | 40 | 0.162 | 0.056 | 3.90E-03 |
| Alkaline phosphatase | CAD | IVW | 42 | -0.145 | 0.034 | 1.54E-05 |
| Alkaline phosphatase | CAD | MR Egger | 42 | -0.270 | 0.040 | 4.33E-08 |
| Alkaline phosphatase | CAD | WM | 42 | -0.224 | 0.029 | 9.08E-15 |
| Aspartate aminotransferase | CAD | IVW | 22 | -0.427 | 0.249 | 8.60E-02 |
| Aspartate aminotransferase | CAD | MR Egger | 22 | -1.277 | 0.734 | 9.71E-02 |
| Aspartate aminotransferase | CAD | WM | 22 | -0.245 | 0.110 | 2.58E-02 |
| Basophil count | CAD | IVW | 22 | -0.030 | 0.051 | 5.61E-01 |
| Basophil count | CAD | MR Egger | 22 | 0.077 | 0.111 | 4.94E-01 |
| Basophil count | CAD | WM | 22 | 0.010 | 0.060 | 8.61E-01 |
| Blood sugar | CAD | IVW | 13 | -0.139 | 0.369 | 7.05E-01 |
| Blood sugar | CAD | MR Egger | 13 | 0.712 | 1.820 | 7.03E-01 |
| Blood sugar | CAD | WM | 13 | 0.090 | 0.114 | 4.29E-01 |
| Blood urea nitrogen | CAD | IVW | 39 | 0.410 | 0.159 | 9.88E-03 |
| Blood urea nitrogen | CAD | MR Egger | 39 | 0.654 | 0.481 | 1.83E-01 |
| Blood urea nitrogen | CAD | WM | 39 | 0.279 | 0.079 | 4.41E-04 |
| Body mass index | CAD | IVW | 59 | 0.179 | 0.107 | 9.41E-02 |
| Body mass index | CAD | MR Egger | 59 | 0.428 | 0.321 | 1.88E-01 |
| Body mass index | CAD | WM | 59 | 0.304 | 0.091 | 8.62E-04 |
| Calcium | CAD | IVW | 12 | 0.113 | 0.084 | 1.78E-01 |
| Calcium | CAD | MR Egger | 12 | 0.380 | 0.337 | 2.85E-01 |
| Calcium | CAD | WM | 12 | 0.185 | 0.096 | 5.36E-02 |
| Chloride | CAD | IVW | 14 | 0.599 | 0.466 | 1.98E-01 |
| Chloride | CAD | MR Egger | 14 | 1.354 | 1.915 | 4.93E-01 |
| Chloride | CAD | WM | 14 | -0.145 | 0.144 | 3.11E-01 |
| C-reactive protein | CAD | IVW | 7 | -0.100 | 0.184 | 5.87E-01 |
| C-reactive protein | CAD | MR Egger | 7 | -0.690 | 0.364 | 1.16E-01 |
| C-reactive protein | CAD | WM | 7 | -0.124 | 0.094 | 1.86E-01 |
| Creatine kinase | CAD | IVW | 32 | -0.022 | 0.089 | 8.05E-01 |
| Creatine kinase | CAD | MR Egger | 32 | -0.005 | 0.251 | 9.86E-01 |
| Creatine kinase | CAD | WM | 32 | -0.091 | 0.090 | 3.11E-01 |
| Diastolic blood pressure | CAD | IVW | 14 | -0.579 | 0.519 | 2.65E-01 |
| Diastolic blood pressure | CAD | MR Egger | 14 | -4.260 | 1.115 | 2.43E-03 |
| Diastolic blood pressure | CAD | WM | 14 | 0.587 | 0.189 | 1.87E-03 |
| Eosinophil count | CAD | IVW | 16 | 0.064 | 0.092 | 4.86E-01 |
| Eosinophil count | CAD | MR Egger | 16 | 0.340 | 0.313 | 2.96E-01 |
| Eosinophil count | CAD | WM | 16 | 0.025 | 0.082 | 7.57E-01 |
| Estimated glomerular filtration rate | CAD | IVW | 54 | -0.190 | 0.134 | 1.56E-01 |
| Estimated glomerular filtration rate | CAD | MR Egger | 54 | -0.562 | 0.421 | 1.88E-01 |
| Estimated glomerular filtration rate | CAD | WM | 54 | -0.194 | 0.075 | 1.03E-02 |
| Gamma glutamyl transferase | CAD | IVW | 53 | -0.139 | 0.100 | 1.65E-01 |
| Gamma glutamyl transferase | CAD | MR Egger | 53 | -0.044 | 0.161 | 7.85E-01 |
| Gamma glutamyl transferase | CAD | WM | 53 | 0.098 | 0.041 | 1.76E-02 |
| Height | CAD | IVW | 424 | 0.000 | 0.002 | 8.23E-01 |
| Height | CAD | MR Egger | 424 | 0.002 | 0.002 | 3.64E-01 |
| Height | CAD | WM | 424 | 0.000 | 0.002 | 9.86E-01 |
| Hematocrit | CAD | IVW | 17 | -0.256 | 0.182 | 1.59E-01 |
| Hematocrit | CAD | MR Egger | 17 | 0.089 | 0.595 | 8.83E-01 |
| Hematocrit | CAD | WM | 17 | -0.064 | 0.133 | 6.28E-01 |
| Hemoglobin | CAD | IVW | 12 | -0.253 | 0.217 | 2.43E-01 |
| Hemoglobin | CAD | MR Egger | 12 | -0.644 | 0.736 | 4.02E-01 |
| Hemoglobin | CAD | WM | 12 | -0.143 | 0.142 | 3.16E-01 |
| Hemoglobin A1c | CAD | IVW | 22 | 0.114 | 0.067 | 8.72E-02 |
| Hemoglobin A1c | CAD | MR Egger | 22 | 0.327 | 0.267 | 2.35E-01 |
| Hemoglobin A1c | CAD | WM | 22 | 0.091 | 0.065 | 1.57E-01 |
| High-density-lipoprotein cholesterol | CAD | IVW | 45 | -0.231 | 0.084 | 5.77E-03 |
| High-density-lipoprotein cholesterol | CAD | MR Egger | 45 | 0.000 | 0.145 | 9.99E-01 |
| High-density-lipoprotein cholesterol | CAD | WM | 45 | -0.017 | 0.039 | 6.62E-01 |
| Lactate dehydrogenase | CAD | IVW | 14 | 0.103 | 0.141 | 4.64E-01 |
| Lactate dehydrogenase | CAD | MR Egger | 14 | -0.056 | 0.181 | 7.62E-01 |
| Lactate dehydrogenase | CAD | WM | 14 | 0.011 | 0.038 | 7.65E-01 |
| Low-density-lipoprotein cholesterol | CAD | IVW | 25 | 0.817 | 0.148 | 3.20E-08 |
| Low-density-lipoprotein cholesterol | CAD | MR Egger | 25 | 0.999 | 0.331 | 6.12E-03 |
| Low-density-lipoprotein cholesterol | CAD | WM | 25 | 0.749 | 0.073 | 1.89E-24 |
| Lymphocyte count | CAD | IVW | 11 | -0.144 | 0.070 | 3.87E-02 |
| Lymphocyte count | CAD | MR Egger | 11 | 0.004 | 0.165 | 9.79E-01 |
| Lymphocyte count | CAD | WM | 11 | -0.112 | 0.094 | 2.36E-01 |
| Mean arterial pressure | CAD | IVW | 26 | -0.063 | 0.321 | 8.44E-01 |
| Mean arterial pressure | CAD | MR Egger | 26 | -3.543 | 0.737 | 6.79E-05 |
| Mean arterial pressure | CAD | WM | 26 | 0.515 | 0.133 | 1.15E-04 |
| Mean corpuscular hemoglobin | CAD | IVW | 82 | -0.145 | 0.063 | 2.09E-02 |
| Mean corpuscular hemoglobin | CAD | MR Egger | 82 | -0.092 | 0.122 | 4.54E-01 |
| Mean corpuscular hemoglobin | CAD | WM | 82 | -0.060 | 0.039 | 1.23E-01 |
| Mean corpuscular volume | CAD | IVW | 83 | -0.062 | 0.057 | 2.73E-01 |
| Mean corpuscular volume | CAD | MR Egger | 83 | -0.173 | 0.113 | 1.31E-01 |
| Mean corpuscular volume | CAD | WM | 83 | -0.062 | 0.039 | 1.16E-01 |
| Menarche | CAD | IVW | 7 | -0.041 | 0.064 | 5.19E-01 |
| Menarche | CAD | MR Egger | 7 | -0.246 | 0.487 | 6.35E-01 |
| Menarche | CAD | WM | 7 | -0.083 | 0.082 | 3.12E-01 |
| Menopause | CAD | IVW | 10 | -0.017 | 0.024 | 4.87E-01 |
| Menopause | CAD | MR Egger | 10 | -0.125 | 0.066 | 9.50E-02 |
| Menopause | CAD | WM | 10 | -0.042 | 0.023 | 6.91E-02 |
| Monocyte count | CAD | IVW | 28 | -0.116 | 0.072 | 1.05E-01 |
| Monocyte count | CAD | MR Egger | 28 | 0.109 | 0.325 | 7.39E-01 |
| Monocyte count | CAD | WM | 28 | -0.086 | 0.065 | 1.91E-01 |
| Neutrophil count | CAD | IVW | 18 | 0.250 | 0.188 | 1.84E-01 |
| Neutrophil count | CAD | MR Egger | 18 | 0.653 | 0.597 | 2.90E-01 |
| Neutrophil count | CAD | WM | 18 | 0.114 | 0.069 | 9.94E-02 |
| Non-albumin protein | CAD | IVW | 45 | -0.034 | 0.050 | 5.00E-01 |
| Non-albumin protein | CAD | MR Egger | 45 | -0.098 | 0.106 | 3.62E-01 |
| Non-albumin protein | CAD | WM | 45 | -0.145 | 0.053 | 6.15E-03 |
| Phosphorus | CAD | IVW | 8 | -0.046 | 0.093 | 6.21E-01 |
| Phosphorus | CAD | MR Egger | 8 | 0.619 | 0.305 | 8.88E-02 |
| Phosphorus | CAD | WM | 8 | -0.074 | 0.100 | 4.62E-01 |
| Platelet count | CAD | IVW | 75 | 0.026 | 0.062 | 6.80E-01 |
| Platelet count | CAD | MR Egger | 75 | -0.023 | 0.149 | 8.80E-01 |
| Platelet count | CAD | WM | 75 | 0.022 | 0.053 | 6.86E-01 |
| Potassium | CAD | IVW | 11 | -0.352 | 0.221 | 1.11E-01 |
| Potassium | CAD | MR Egger | 11 | -0.770 | 0.996 | 4.59E-01 |
| Potassium | CAD | WM | 11 | -0.192 | 0.172 | 2.65E-01 |
| Prothrombin time | CAD | IVW | 9 | -0.026 | 0.044 | 5.61E-01 |
| Prothrombin time | CAD | MR Egger | 9 | -0.013 | 0.063 | 8.45E-01 |
| Prothrombin time | CAD | WM | 9 | -0.020 | 0.042 | 6.39E-01 |
| Pulse pressure | CAD | IVW | 12 | -0.297 | 0.681 | 6.63E-01 |
| Pulse pressure | CAD | MR Egger | 12 | 1.207 | 4.042 | 7.71E-01 |
| Pulse pressure | CAD | WM | 12 | 0.265 | 0.211 | 2.09E-01 |
| Red blood cell count | CAD | IVW | 45 | 0.062 | 0.120 | 6.05E-01 |
| Red blood cell count | CAD | MR Egger | 45 | 0.168 | 0.242 | 4.92E-01 |
| Red blood cell count | CAD | WM | 45 | 0.098 | 0.061 | 1.08E-01 |
| Serum creatinine | CAD | IVW | 49 | 0.316 | 0.136 | 1.99E-02 |
| Serum creatinine | CAD | MR Egger | 49 | 0.533 | 0.433 | 2.24E-01 |
| Serum creatinine | CAD | WM | 49 | 0.209 | 0.079 | 8.17E-03 |
| Sodium | CAD | IVW | 11 | 0.464 | 0.624 | 4.58E-01 |
| Sodium | CAD | MR Egger | 11 | -2.413 | 3.186 | 4.68E-01 |
| Sodium | CAD | WM | 11 | -0.243 | 0.176 | 1.66E-01 |
| Systolic blood pressure | CAD | IVW | 18 | -0.155 | 0.419 | 7.11E-01 |
| Systolic blood pressure | CAD | MR Egger | 18 | -4.367 | 1.003 | 4.92E-04 |
| Systolic blood pressure | CAD | WM | 18 | 0.492 | 0.164 | 2.69E-03 |
| Total bilirubin | CAD | IVW | 16 | -0.001 | 0.037 | 9.75E-01 |
| Total bilirubin | CAD | MR Egger | 16 | 0.049 | 0.048 | 3.28E-01 |
| Total bilirubin | CAD | WM | 16 | 0.033 | 0.027 | 2.27E-01 |
| Total cholesterol | CAD | IVW | 40 | 0.615 | 0.099 | 4.25E-10 |
| Total cholesterol | CAD | MR Egger | 40 | 1.099 | 0.238 | 4.25E-05 |
| Total cholesterol | CAD | WM | 40 | 0.614 | 0.089 | 5.96E-12 |
| Total protein | CAD | IVW | 31 | -0.073 | 0.115 | 5.25E-01 |
| Total protein | CAD | MR Egger | 31 | -0.224 | 0.262 | 4.00E-01 |
| Total protein | CAD | WM | 31 | -0.155 | 0.075 | 3.86E-02 |
| Triglyceride | CAD | IVW | 31 | 0.212 | 0.052 | 4.07E-05 |
| Triglyceride | CAD | MR Egger | 31 | 0.275 | 0.081 | 2.08E-03 |
| Triglyceride | CAD | WM | 31 | 0.270 | 0.042 | 1.41E-10 |
| Uric acid | CAD | IVW | 41 | 0.092 | 0.098 | 3.48E-01 |
| Uric acid | CAD | MR Egger | 41 | 0.166 | 0.168 | 3.30E-01 |
| Uric acid | CAD | WM | 41 | 0.160 | 0.039 | 4.15E-05 |
| White blood cell count | CAD | IVW | 27 | 0.080 | 0.209 | 7.03E-01 |
| White blood cell count | CAD | MR Egger | 27 | 0.363 | 0.809 | 6.57E-01 |
| White blood cell count | CAD | WM | 27 | -0.061 | 0.092 | 5.10E-01 |
| T2D | Activated partial thromboplastin time | IVW | 82 | -0.016 | 0.016 | 3.19E-01 |
| T2D | Activated partial thromboplastin time | MR Egger | 82 | -0.018 | 0.035 | 6.06E-01 |
| T2D | Activated partial thromboplastin time | WM | 82 | -0.011 | 0.017 | 5.12E-01 |
| T2D | Alanine aminotransferase | IVW | 82 | -0.031 | 0.008 | 5.81E-05 |
| T2D | Alanine aminotransferase | MR Egger | 82 | -0.075 | 0.016 | 1.53E-05 |
| T2D | Alanine aminotransferase | WM | 82 | -0.044 | 0.009 | 1.75E-06 |
| T2D | Albumin | IVW | 82 | 0.010 | 0.013 | 4.27E-01 |
| T2D | Albumin | MR Egger | 82 | 0.014 | 0.028 | 6.11E-01 |
| T2D | Albumin | WM | 82 | 0.022 | 0.010 | 2.62E-02 |
| T2D | Albumin/globulin ratio | IVW | 82 | 0.014 | 0.011 | 1.95E-01 |
| T2D | Albumin/globulin ratio | MR Egger | 82 | 0.026 | 0.024 | 2.70E-01 |
| T2D | Albumin/globulin ratio | WM | 82 | 0.024 | 0.011 | 2.49E-02 |
| T2D | Alkaline phosphatase | IVW | 82 | -0.031 | 0.043 | 4.72E-01 |
| T2D | Alkaline phosphatase | MR Egger | 82 | 0.070 | 0.096 | 4.67E-01 |
| T2D | Alkaline phosphatase | WM | 82 | 0.005 | 0.010 | 6.57E-01 |
| T2D | Aspartate aminotransferase | IVW | 82 | -0.043 | 0.008 | 1.42E-08 |
| T2D | Aspartate aminotransferase | MR Egger | 82 | -0.061 | 0.017 | 5.71E-04 |
| T2D | Aspartate aminotransferase | WM | 82 | -0.043 | 0.008 | 4.68E-08 |
| T2D | Basophil count | IVW | 82 | -0.005 | 0.009 | 5.73E-01 |
| T2D | Basophil count | MR Egger | 82 | 0.011 | 0.021 | 6.08E-01 |
| T2D | Basophil count | WM | 82 | 0.005 | 0.013 | 7.29E-01 |
| T2D | Blood sugar | IVW | 82 | 0.181 | 0.012 | 1.15E-48 |
| T2D | Blood sugar | MR Egger | 82 | 0.185 | 0.028 | 2.41E-09 |
| T2D | Blood sugar | WM | 82 | 0.175 | 0.013 | 2.39E-43 |
| T2D | Blood urea nitrogen | IVW | 82 | 0.010 | 0.009 | 2.61E-01 |
| T2D | Blood urea nitrogen | MR Egger | 82 | 0.019 | 0.021 | 3.56E-01 |
| T2D | Blood urea nitrogen | WM | 82 | 0.013 | 0.009 | 1.18E-01 |
| T2D | Body mass index | IVW | 82 | -0.100 | 0.021 | 2.56E-06 |
| T2D | Body mass index | MR Egger | 82 | -0.181 | 0.046 | 1.91E-04 |
| T2D | Body mass index | WM | 82 | -0.157 | 0.010 | 8.48E-54 |
| T2D | Calcium | IVW | 82 | 0.003 | 0.009 | 7.75E-01 |
| T2D | Calcium | MR Egger | 82 | -0.007 | 0.020 | 7.30E-01 |
| T2D | Calcium | WM | 82 | -0.013 | 0.012 | 2.80E-01 |
| T2D | Chloride | IVW | 82 | -0.059 | 0.009 | 1.05E-10 |
| T2D | Chloride | MR Egger | 82 | -0.074 | 0.020 | 4.90E-04 |
| T2D | Chloride | WM | 82 | -0.049 | 0.010 | 5.40E-07 |
| T2D | C-reactive protein | IVW | 82 | -0.025 | 0.010 | 1.16E-02 |
| T2D | C-reactive protein | MR Egger | 82 | -0.013 | 0.022 | 5.41E-01 |
| T2D | C-reactive protein | WM | 82 | -0.020 | 0.012 | 9.33E-02 |
| T2D | Creatine kinase | IVW | 82 | -0.015 | 0.010 | 1.50E-01 |
| T2D | Creatine kinase | MR Egger | 82 | -0.059 | 0.022 | 7.84E-03 |
| T2D | Creatine kinase | WM | 82 | -0.034 | 0.010 | 5.81E-04 |
| T2D | Diastolic blood pressure | IVW | 82 | -0.046 | 0.008 | 5.13E-09 |
| T2D | Diastolic blood pressure | MR Egger | 82 | -0.067 | 0.017 | 2.45E-04 |
| T2D | Diastolic blood pressure | WM | 82 | -0.054 | 0.010 | 4.80E-08 |
| T2D | Eosinophil count | IVW | 82 | -0.017 | 0.008 | 4.03E-02 |
| T2D | Eosinophil count | MR Egger | 82 | -0.035 | 0.018 | 5.88E-02 |
| T2D | Eosinophil count | WM | 82 | -0.015 | 0.012 | 2.12E-01 |
| T2D | Estimated glomerular filtration rate | IVW | 82 | 0.017 | 0.010 | 9.48E-02 |
| T2D | Estimated glomerular filtration rate | MR Egger | 82 | 0.028 | 0.022 | 2.01E-01 |
| T2D | Estimated glomerular filtration rate | WM | 82 | 0.016 | 0.009 | 6.13E-02 |
| T2D | Gamma glutamyl transferase | IVW | 82 | -0.036 | 0.013 | 3.96E-03 |
| T2D | Gamma glutamyl transferase | MR Egger | 82 | -0.044 | 0.028 | 1.21E-01 |
| T2D | Gamma glutamyl transferase | WM | 82 | -0.037 | 0.010 | 1.64E-04 |
| T2D | Height | IVW | 82 | -0.019 | 0.016 | 2.33E-01 |
| T2D | Height | MR Egger | 82 | -0.033 | 0.035 | 3.44E-01 |
| T2D | Height | WM | 82 | -0.017 | 0.008 | 2.47E-02 |
| T2D | Hematocrit | IVW | 82 | -0.053 | 0.011 | 3.46E-06 |
| T2D | Hematocrit | MR Egger | 82 | -0.032 | 0.025 | 2.10E-01 |
| T2D | Hematocrit | WM | 82 | -0.046 | 0.009 | 9.77E-07 |
| T2D | Hemoglobin | IVW | 82 | -0.049 | 0.011 | 2.08E-05 |
| T2D | Hemoglobin | MR Egger | 82 | -0.023 | 0.025 | 3.63E-01 |
| T2D | Hemoglobin | WM | 82 | -0.041 | 0.010 | 1.82E-05 |
| T2D | Hemoglobin A1c | IVW | 82 | 0.333 | 0.014 | 2.55E-128 |
| T2D | Hemoglobin A1c | MR Egger | 82 | 0.375 | 0.030 | 3.06E-20 |
| T2D | Hemoglobin A1c | WM | 82 | 0.330 | 0.018 | 2.12E-78 |
| T2D | High-density-lipoprotein cholesterol | IVW | 82 | 0.035 | 0.012 | 2.70E-03 |
| T2D | High-density-lipoprotein cholesterol | MR Egger | 82 | 0.084 | 0.026 | 1.48E-03 |
| T2D | High-density-lipoprotein cholesterol | WM | 82 | 0.059 | 0.012 | 1.81E-06 |
| T2D | Lactate dehydrogenase | IVW | 82 | -0.017 | 0.008 | 2.67E-02 |
| T2D | Lactate dehydrogenase | MR Egger | 82 | -0.062 | 0.017 | 3.79E-04 |
| T2D | Lactate dehydrogenase | WM | 82 | -0.016 | 0.009 | 8.43E-02 |
| T2D | Low-density-lipoprotein cholesterol | IVW | 82 | 0.021 | 0.011 | 5.98E-02 |
| T2D | Low-density-lipoprotein cholesterol | MR Egger | 82 | 0.007 | 0.025 | 7.71E-01 |
| T2D | Low-density-lipoprotein cholesterol | WM | 82 | 0.018 | 0.012 | 1.28E-01 |
| T2D | Lymphocyte count | IVW | 82 | -0.013 | 0.011 | 2.33E-01 |
| T2D | Lymphocyte count | MR Egger | 82 | -0.017 | 0.025 | 4.84E-01 |
| T2D | Lymphocyte count | WM | 82 | -0.021 | 0.014 | 1.17E-01 |
| T2D | Mean arterial pressure | IVW | 82 | -0.034 | 0.008 | 4.49E-05 |
| T2D | Mean arterial pressure | MR Egger | 82 | -0.055 | 0.018 | 3.43E-03 |
| T2D | Mean arterial pressure | WM | 82 | -0.049 | 0.010 | 4.50E-07 |
| T2D | Mean corpuscular hemoglobin | IVW | 82 | -0.017 | 0.011 | 1.13E-01 |
| T2D | Mean corpuscular hemoglobin | MR Egger | 82 | 0.000 | 0.024 | 9.90E-01 |
| T2D | Mean corpuscular hemoglobin | WM | 82 | 0.009 | 0.010 | 3.78E-01 |
| T2D | Mean corpuscular volume | IVW | 82 | -0.022 | 0.011 | 4.55E-02 |
| T2D | Mean corpuscular volume | MR Egger | 82 | -0.008 | 0.025 | 7.54E-01 |
| T2D | Mean corpuscular volume | WM | 82 | 0.001 | 0.011 | 9.56E-01 |
| T2D | Menarche | IVW | 94 | -0.027 | 0.016 | 9.43E-02 |
| T2D | Menarche | MR Egger | 94 | -0.012 | 0.036 | 7.46E-01 |
| T2D | Menarche | WM | 94 | 0.005 | 0.022 | 8.33E-01 |
| T2D | Menopause | IVW | 94 | 0.064 | 0.041 | 1.19E-01 |
| T2D | Menopause | MR Egger | 94 | 0.126 | 0.091 | 1.72E-01 |
| T2D | Menopause | WM | 94 | 0.092 | 0.060 | 1.25E-01 |
| T2D | Monocyte count | IVW | 82 | -0.036 | 0.010 | 4.52E-04 |
| T2D | Monocyte count | MR Egger | 82 | -0.016 | 0.022 | 4.67E-01 |
| T2D | Monocyte count | WM | 82 | -0.041 | 0.014 | 2.73E-03 |
| T2D | Neutrophil count | IVW | 82 | -0.019 | 0.012 | 1.16E-01 |
| T2D | Neutrophil count | MR Egger | 82 | 0.016 | 0.026 | 5.47E-01 |
| T2D | Neutrophil count | WM | 82 | -0.007 | 0.013 | 5.58E-01 |
| T2D | Non-albumin protein | IVW | 82 | -0.011 | 0.009 | 2.29E-01 |
| T2D | Non-albumin protein | MR Egger | 82 | -0.027 | 0.020 | 1.90E-01 |
| T2D | Non-albumin protein | WM | 82 | -0.013 | 0.010 | 2.00E-01 |
| T2D | Phosphorus | IVW | 82 | 0.022 | 0.010 | 2.24E-02 |
| T2D | Phosphorus | MR Egger | 82 | 0.031 | 0.021 | 1.43E-01 |
| T2D | Phosphorus | WM | 82 | 0.019 | 0.016 | 2.35E-01 |
| T2D | Platelet count | IVW | 82 | 0.001 | 0.011 | 9.10E-01 |
| T2D | Platelet count | MR Egger | 82 | -0.027 | 0.025 | 2.80E-01 |
| T2D | Platelet count | WM | 82 | 0.007 | 0.010 | 4.88E-01 |
| T2D | Potassium | IVW | 82 | 0.038 | 0.007 | 2.71E-07 |
| T2D | Potassium | MR Egger | 82 | 0.055 | 0.016 | 1.12E-03 |
| T2D | Potassium | WM | 82 | 0.041 | 0.009 | 1.59E-05 |
| T2D | Prothrombin time | IVW | 82 | -0.006 | 0.009 | 4.97E-01 |
| T2D | Prothrombin time | MR Egger | 82 | -0.028 | 0.021 | 1.75E-01 |
| T2D | Prothrombin time | WM | 82 | -0.006 | 0.013 | 6.38E-01 |
| T2D | Pulse pressure | IVW | 82 | 0.024 | 0.007 | 8.26E-04 |
| T2D | Pulse pressure | MR Egger | 82 | 0.016 | 0.016 | 3.25E-01 |
| T2D | Pulse pressure | WM | 82 | 0.009 | 0.010 | 3.29E-01 |
| T2D | Red blood cell count | IVW | 82 | -0.037 | 0.013 | 5.06E-03 |
| T2D | Red blood cell count | MR Egger | 82 | -0.026 | 0.029 | 3.73E-01 |
| T2D | Red blood cell count | WM | 82 | -0.046 | 0.010 | 3.78E-06 |
| T2D | Serum creatinine | IVW | 82 | -0.008 | 0.010 | 4.42E-01 |
| T2D | Serum creatinine | MR Egger | 82 | -0.018 | 0.023 | 4.15E-01 |
| T2D | Serum creatinine | WM | 82 | -0.015 | 0.009 | 1.10E-01 |
| T2D | Sodium | IVW | 82 | -0.043 | 0.009 | 1.20E-06 |
| T2D | Sodium | MR Egger | 82 | -0.062 | 0.020 | 2.23E-03 |
| T2D | Sodium | WM | 82 | -0.049 | 0.010 | 1.43E-06 |
| T2D | Systolic blood pressure | IVW | 82 | -0.012 | 0.008 | 1.59E-01 |
| T2D | Systolic blood pressure | MR Egger | 82 | -0.031 | 0.018 | 9.82E-02 |
| T2D | Systolic blood pressure | WM | 82 | -0.031 | 0.009 | 6.28E-04 |
| T2D | Total bilirubin | IVW | 82 | -0.002 | 0.008 | 7.69E-01 |
| T2D | Total bilirubin | MR Egger | 82 | 0.000 | 0.017 | 9.83E-01 |
| T2D | Total bilirubin | WM | 82 | 0.008 | 0.010 | 4.10E-01 |
| T2D | Total cholesterol | IVW | 82 | 0.019 | 0.011 | 1.00E-01 |
| T2D | Total cholesterol | MR Egger | 82 | 0.033 | 0.025 | 1.89E-01 |
| T2D | Total cholesterol | WM | 82 | 0.030 | 0.009 | 8.10E-04 |
| T2D | Total protein | IVW | 82 | -0.004 | 0.010 | 7.01E-01 |
| T2D | Total protein | MR Egger | 82 | -0.012 | 0.023 | 6.12E-01 |
| T2D | Total protein | WM | 82 | -0.004 | 0.010 | 6.64E-01 |
| T2D | Triglyceride | IVW | 82 | -0.028 | 0.017 | 1.02E-01 |
| T2D | Triglyceride | MR Egger | 82 | -0.029 | 0.038 | 4.58E-01 |
| T2D | Triglyceride | WM | 82 | -0.021 | 0.011 | 6.06E-02 |
| T2D | Uric acid | IVW | 82 | -0.044 | 0.011 | 2.66E-05 |
| T2D | Uric acid | MR Egger | 82 | -0.071 | 0.023 | 3.05E-03 |
| T2D | Uric acid | WM | 82 | -0.058 | 0.010 | 4.42E-09 |
| T2D | White blood cell count | IVW | 82 | -0.030 | 0.011 | 6.12E-03 |
| T2D | White blood cell count | MR Egger | 82 | -0.001 | 0.024 | 9.67E-01 |
| T2D | White blood cell count | WM | 82 | -0.013 | 0.011 | 2.04E-01 |

Supplementary Table 3. Horizontal pleiotropy tested by MR-Egger method

| Exposure | Outcome | Egger_intercept | SE | P |
| --- | --- | --- | --- | --- |
| Activated partial thromboplastin time | T2D | -0.017 | 0.010 | 0.118 |
| Alanine aminotransferase | T2D | -0.013 | 0.016 | 0.436 |
| Albumin | T2D | 0.021 | 0.015 | 0.180 |
| Albumin/globulin ratio | T2D | -0.006 | 0.006 | 0.331 |
| Alkaline phosphatase | T2D | 0.004 | 0.003 | 0.278 |
| Aspartate aminotransferase | T2D | -0.015 | 0.013 | 0.247 |
| Basophil count | T2D | -0.004 | 0.007 | 0.555 |
| Blood sugar | T2D | 0.118 | 0.095 | 0.233 |
| Blood urea nitrogen | T2D | -0.003 | 0.014 | 0.822 |
| Body mass index | T2D | 0.010 | 0.028 | 0.709 |
| Calcium | T2D | -0.028 | 0.018 | 0.158 |
| Chloride | T2D | -0.061 | 0.095 | 0.537 |
| C-reactive protein | T2D | -0.019 | 0.023 | 0.450 |
| Creatine kinase | T2D | 0.024 | 0.009 | 0.010 |
| Diastolic blood pressure | T2D | -0.047 | 0.016 | 0.012 |
| Eosinophil count | T2D | -0.007 | 0.012 | 0.556 |
| Estimated glomerular filtration rate | T2D | -0.008 | 0.010 | 0.430 |
| Gamma glutamyl transferase | T2D | 0.004 | 0.004 | 0.413 |
| Height | T2D | -0.002 | 0.001 | 0.087 |
| Hematocrit | T2D | 0.008 | 0.012 | 0.500 |
| Hemoglobin | T2D | 0.014 | 0.012 | 0.288 |
| Hemoglobin A1c | T2D | -0.093 | 0.058 | 0.125 |
| High-density-lipoprotein cholesterol | T2D | 0.000 | 0.008 | 0.992 |
| Lactate dehydrogenase | T2D | 0.017 | 0.011 | 0.146 |
| Low-density-lipoprotein cholesterol | T2D | -0.007 | 0.007 | 0.315 |
| Lymphocyte count | T2D | -0.001 | 0.014 | 0.963 |
| Mean arterial pressure | T2D | -0.028 | 0.013 | 0.040 |
| Mean corpuscular hemoglobin | T2D | -0.005 | 0.003 | 0.129 |
| Mean corpuscular volume | T2D | -0.008 | 0.003 | 0.013 |
| Menarche | T2D | 0.024 | 0.024 | 0.373 |
| Menopause | T2D | 0.014 | 0.018 | 0.467 |
| Monocyte count | T2D | -0.026 | 0.009 | 0.005 |
| Neutrophil count | T2D | -0.021 | 0.013 | 0.104 |
| Non-albumin protein | T2D | 0.003 | 0.004 | 0.444 |
| Phosphorus | T2D | 0.008 | 0.018 | 0.668 |
| Platelet count | T2D | 0.002 | 0.005 | 0.746 |
| Potassium | T2D | 0.021 | 0.013 | 0.140 |
| Prothrombin time | T2D | 0.014 | 0.010 | 0.216 |
| Pulse pressure | T2D | -0.014 | 0.029 | 0.632 |
| Red blood cell count | T2D | -0.003 | 0.005 | 0.587 |
| Serum creatinine | T2D | 0.012 | 0.009 | 0.203 |
| Sodium | T2D | 0.002 | 0.042 | 0.969 |
| Systolic blood pressure | T2D | -0.029 | 0.015 | 0.075 |
| Total bilirubin | T2D | 0.000 | 0.006 | 0.964 |
| Total cholesterol | T2D | -0.006 | 0.006 | 0.341 |
| Total protein | T2D | 0.008 | 0.010 | 0.431 |
| Triglyceride | T2D | 0.001 | 0.008 | 0.916 |
| Uric acid | T2D | -0.002 | 0.006 | 0.676 |
| White blood cell count | T2D | -0.022 | 0.011 | 0.054 |
| Activated partial thromboplastin time | CAD | 0.002 | 0.024 | 0.949 |
| Alanine aminotransferase | CAD | 0.077 | 0.027 | 0.010 |
| Albumin | CAD | -0.001 | 0.011 | 0.937 |
| Albumin/globulin ratio | CAD | -0.007 | 0.006 | 0.245 |
| Alkaline phosphatase | CAD | 0.015 | 0.003 | 0.000 |
| Aspartate aminotransferase | CAD | 0.033 | 0.027 | 0.233 |
| Basophil count | CAD | -0.007 | 0.006 | 0.290 |
| Blood sugar | CAD | -0.036 | 0.076 | 0.642 |
| Blood urea nitrogen | CAD | -0.009 | 0.016 | 0.595 |
| Body mass index | CAD | -0.008 | 0.010 | 0.416 |
| Calcium | CAD | -0.012 | 0.014 | 0.432 |
| Chloride | CAD | -0.023 | 0.057 | 0.691 |
| C-reactive protein | CAD | 0.045 | 0.025 | 0.132 |
| Creatine kinase | CAD | -0.001 | 0.009 | 0.941 |
| Diastolic blood pressure | CAD | 0.122 | 0.035 | 0.004 |
| Eosinophil count | CAD | -0.016 | 0.017 | 0.372 |
| Estimated glomerular filtration rate | CAD | 0.013 | 0.014 | 0.356 |
| Gamma glutamyl transferase | CAD | -0.006 | 0.009 | 0.460 |
| Height | CAD | -0.002 | 0.001 | 0.041 |
| Hematocrit | CAD | -0.013 | 0.022 | 0.551 |
| Hemoglobin | CAD | 0.015 | 0.028 | 0.589 |
| Hemoglobin A1c | CAD | -0.014 | 0.017 | 0.419 |
| High-density-lipoprotein cholesterol | CAD | -0.021 | 0.011 | 0.061 |
| Lactate dehydrogenase | CAD | 0.023 | 0.017 | 0.203 |
| Low-density-lipoprotein cholesterol | CAD | -0.012 | 0.019 | 0.545 |
| Lymphocyte count | CAD | -0.008 | 0.008 | 0.349 |
| Mean arterial pressure | CAD | 0.113 | 0.023 | 0.000 |
| Mean corpuscular hemoglobin | CAD | -0.003 | 0.007 | 0.613 |
| Mean corpuscular volume | CAD | 0.007 | 0.006 | 0.263 |
| Menarche | CAD | 0.013 | 0.031 | 0.689 |
| Menopause | CAD | 0.024 | 0.014 | 0.121 |
| Monocyte count | CAD | -0.011 | 0.015 | 0.483 |
| Neutrophil count | CAD | -0.024 | 0.033 | 0.486 |
| Non-albumin protein | CAD | 0.003 | 0.005 | 0.497 |
| Phosphorus | CAD | -0.045 | 0.020 | 0.066 |
| Platelet count | CAD | 0.002 | 0.006 | 0.723 |
| Potassium | CAD | 0.014 | 0.033 | 0.676 |
| Prothrombin time | CAD | -0.002 | 0.007 | 0.765 |
| Pulse pressure | CAD | -0.047 | 0.124 | 0.713 |
| Red blood cell count | CAD | -0.006 | 0.012 | 0.616 |
| Serum creatinine | CAD | -0.008 | 0.015 | 0.600 |
| Sodium | CAD | 0.095 | 0.103 | 0.381 |
| Systolic blood pressure | CAD | 0.147 | 0.034 | 0.000 |
| Total bilirubin | CAD | -0.009 | 0.006 | 0.147 |
| Total cholesterol | CAD | -0.022 | 0.010 | 0.033 |
| Total protein | CAD | 0.007 | 0.011 | 0.526 |
| Triglyceride | CAD | -0.005 | 0.005 | 0.324 |
| Uric acid | CAD | -0.006 | 0.011 | 0.590 |
| White blood cell count | CAD | -0.011 | 0.030 | 0.720 |
| T2D | Activated partial thromboplastin time | 0.000 | 0.003 | 0.936 |
| T2D | Alanine aminotransferase | 0.005 | 0.002 | 0.003 |
| T2D | Albumin | 0.000 | 0.003 | 0.864 |
| T2D | Albumin/globulin ratio | -0.001 | 0.002 | 0.555 |
| T2D | Alkaline phosphatase | -0.011 | 0.009 | 0.240 |
| T2D | Aspartate aminotransferase | 0.002 | 0.002 | 0.255 |
| T2D | Basophil count | -0.002 | 0.002 | 0.391 |
| T2D | Blood sugar | 0.000 | 0.003 | 0.868 |
| T2D | Blood urea nitrogen | -0.001 | 0.002 | 0.637 |
| T2D | Body mass index | 0.008 | 0.004 | 0.053 |
| T2D | Calcium | 0.001 | 0.002 | 0.596 |
| T2D | Chloride | 0.002 | 0.002 | 0.419 |
| T2D | C-reactive protein | -0.001 | 0.002 | 0.561 |
| T2D | Creatine kinase | 0.005 | 0.002 | 0.024 |
| T2D | Diastolic blood pressure | 0.002 | 0.002 | 0.187 |
| T2D | Eosinophil count | 0.002 | 0.002 | 0.271 |
| T2D | Estimated glomerular filtration rate | -0.001 | 0.002 | 0.549 |
| T2D | Gamma glutamyl transferase | 0.001 | 0.003 | 0.758 |
| T2D | Height | 0.002 | 0.003 | 0.646 |
| T2D | Hematocrit | -0.002 | 0.002 | 0.355 |
| T2D | Hemoglobin | -0.003 | 0.002 | 0.261 |
| T2D | Hemoglobin A1c | -0.004 | 0.003 | 0.124 |
| T2D | High-density-lipoprotein cholesterol | -0.005 | 0.002 | 0.036 |
| T2D | Lactate dehydrogenase | 0.005 | 0.002 | 0.004 |
| T2D | Low-density-lipoprotein cholesterol | 0.001 | 0.002 | 0.536 |
| T2D | Lymphocyte count | 0.000 | 0.002 | 0.851 |
| T2D | Mean arterial pressure | 0.002 | 0.002 | 0.195 |
| T2D | Mean corpuscular hemoglobin | -0.002 | 0.002 | 0.418 |
| T2D | Mean corpuscular volume | -0.001 | 0.002 | 0.513 |
| T2D | Menarche | -0.002 | 0.003 | 0.632 |
| T2D | Menopause | -0.006 | 0.008 | 0.450 |
| T2D | Monocyte count | -0.002 | 0.002 | 0.343 |
| T2D | Neutrophil count | -0.004 | 0.002 | 0.143 |
| T2D | Non-albumin protein | 0.002 | 0.002 | 0.385 |
| T2D | Phosphorus | -0.001 | 0.002 | 0.615 |
| T2D | Platelet count | 0.003 | 0.002 | 0.205 |
| T2D | Potassium | -0.002 | 0.002 | 0.241 |
| T2D | Prothrombin time | 0.002 | 0.002 | 0.237 |
| T2D | Pulse pressure | 0.001 | 0.002 | 0.567 |
| T2D | Red blood cell count | -0.001 | 0.003 | 0.685 |
| T2D | Serum creatinine | 0.001 | 0.002 | 0.596 |
| T2D | Sodium | 0.002 | 0.002 | 0.283 |
| T2D | Systolic blood pressure | 0.002 | 0.002 | 0.249 |
| T2D | Total bilirubin | 0.000 | 0.002 | 0.863 |
| T2D | Total cholesterol | -0.002 | 0.002 | 0.514 |
| T2D | Total protein | 0.001 | 0.002 | 0.707 |
| T2D | Triglyceride | 0.000 | 0.004 | 0.989 |
| T2D | Uric acid | 0.003 | 0.002 | 0.201 |
| T2D | White blood cell count | -0.003 | 0.002 | 0.181 |

Supplementary Table 4. Significance of the effect of traits/T2D after FDR correction

| Trait | Effect of trait on T2D (BH-adjusted P) | Effect of trait on T2D (significance) | Effect of T2D on trait (BH-adjusted P) | Effect of T2D on trait (significance) | Effect of trait on CAD (BH-adjusted P) | Effect of trait on CAD (significance) |
| --- | --- | --- | --- | --- | --- | --- |
| Hemoglobin A1c | 7.41E-10 | TRUE | 1.30E-126 | TRUE | 3.29E-01 | FALSE |
| Blood sugar | 7.28E-05 | TRUE | 2.94E-47 | TRUE | 7.92E-01 | FALSE |
| Red blood cell count | 3.98E-02 | TRUE | 1.43E-02 | TRUE | 7.80E-01 | FALSE |
| Monocyte count | 6.69E-02 | FALSE | 1.65E-03 | TRUE | 3.40E-01 | FALSE |
| Alkaline phosphatase | 1.11E-01 | FALSE | 5.59E-01 | FALSE | 2.52E-04 | TRUE |
| White blood cell count | 1.53E-01 | FALSE | 1.64E-02 | TRUE | 7.92E-01 | FALSE |
| Platelet count | 3.58E-01 | FALSE | 9.10E-01 | FALSE | 7.92E-01 | FALSE |
| Neutrophil count | 4.85E-01 | FALSE | 1.90E-01 | FALSE | 4.28E-01 | FALSE |
| Activated partial thromboplastin time | 4.85E-01 | FALSE | 4.07E-01 | FALSE | 4.63E-02 | TRUE |
| Albumin | 4.85E-01 | FALSE | 5.31E-01 | FALSE | 7.56E-01 | FALSE |
| Total protein | 5.88E-01 | FALSE | 7.77E-01 | FALSE | 7.56E-01 | FALSE |
| Sodium | 6.23E-01 | FALSE | 8.77E-06 | TRUE | 7.56E-01 | FALSE |
| Gamma glutamyl transferase | 7.26E-01 | FALSE | 1.19E-02 | TRUE | 4.25E-01 | FALSE |
| Non-albumin protein | 7.26E-01 | FALSE | 3.13E-01 | FALSE | 7.56E-01 | FALSE |
| Potassium | 8.64E-01 | FALSE | 2.30E-06 | TRUE | 3.40E-01 | FALSE |
| Hematocrit | 8.64E-01 | FALSE | 1.96E-05 | TRUE | 4.25E-01 | FALSE |
| Lymphocyte count | 8.64E-01 | FALSE | 3.13E-01 | FALSE | 1.72E-01 | FALSE |
| Chloride | 8.74E-01 | FALSE | 1.78E-09 | TRUE | 4.42E-01 | FALSE |
| Systolic blood pressure | 9.07E-01 | FALSE | 2.39E-01 | FALSE | 7.92E-01 | FALSE |
| Albumin/globulin ratio | 9.07E-01 | FALSE | 2.85E-01 | FALSE | 8.61E-01 | FALSE |
| Hemoglobin | 9.29E-01 | FALSE | 1.06E-04 | TRUE | 5.18E-01 | FALSE |
| Mean arterial pressure | 9.29E-01 | FALSE | 1.91E-04 | TRUE | 8.61E-01 | FALSE |
| Alanine aminotransferase | 9.29E-01 | FALSE | 2.28E-04 | TRUE | 1.72E-01 | FALSE |
| Low-density-lipoprotein cholesterol | 9.29E-01 | FALSE | 1.22E-01 | FALSE | 7.85E-07 | TRUE |
| Mean corpuscular hemoglobin | 9.29E-01 | FALSE | 1.90E-01 | FALSE | 1.14E-01 | FALSE |
| Menarche | 9.33E-01 | FALSE | 1.79E-01 | FALSE | 7.56E-01 | FALSE |
| Diastolic blood pressure | 9.69E-01 | FALSE | 6.54E-08 | TRUE | 5.35E-01 | FALSE |
| Aspartate aminotransferase | 9.69E-01 | FALSE | 1.44E-07 | TRUE | 3.29E-01 | FALSE |
| Body mass index | 9.69E-01 | FALSE | 1.63E-05 | TRUE | 3.29E-01 | FALSE |
| Uric acid | 9.69E-01 | FALSE | 1.23E-04 | TRUE | 6.57E-01 | FALSE |
| Pulse pressure | 9.69E-01 | FALSE | 2.81E-03 | TRUE | 7.92E-01 | FALSE |
| High-density-lipoprotein cholesterol | 9.69E-01 | FALSE | 8.60E-03 | TRUE | 4.71E-02 | TRUE |
| C-reactive protein | 9.69E-01 | FALSE | 2.97E-02 | TRUE | 7.77E-01 | FALSE |
| Phosphorus | 9.69E-01 | FALSE | 5.43E-02 | FALSE | 7.80E-01 | FALSE |
| Lactate dehydrogenase | 9.69E-01 | FALSE | 6.19E-02 | FALSE | 7.56E-01 | FALSE |
| Eosinophil count | 9.69E-01 | FALSE | 8.93E-02 | FALSE | 7.56E-01 | FALSE |
| Mean corpuscular volume | 9.69E-01 | FALSE | 9.67E-02 | FALSE | 5.35E-01 | FALSE |
| Total cholesterol | 9.69E-01 | FALSE | 1.79E-01 | FALSE | 2.08E-08 | TRUE |
| Triglyceride | 9.69E-01 | FALSE | 1.79E-01 | FALSE | 4.98E-04 | TRUE |
| Estimated glomerular filtration rate | 9.69E-01 | FALSE | 1.79E-01 | FALSE | 4.25E-01 | FALSE |
| Menopause | 9.69E-01 | FALSE | 1.90E-01 | FALSE | 7.56E-01 | FALSE |
| Creatine kinase | 9.69E-01 | FALSE | 2.31E-01 | FALSE | 8.61E-01 | FALSE |
| Height | 9.69E-01 | FALSE | 3.13E-01 | FALSE | 8.61E-01 | FALSE |
| Blood urea nitrogen | 9.69E-01 | FALSE | 3.41E-01 | FALSE | 6.92E-02 | FALSE |
| Serum creatinine | 9.69E-01 | FALSE | 5.37E-01 | FALSE | 1.14E-01 | FALSE |
| Prothrombin time | 9.69E-01 | FALSE | 5.76E-01 | FALSE | 7.64E-01 | FALSE |
| Basophil count | 9.69E-01 | FALSE | 6.49E-01 | FALSE | 7.64E-01 | FALSE |
| Calcium | 9.69E-01 | FALSE | 8.07E-01 | FALSE | 4.28E-01 | FALSE |
| Total bilirubin | 9.69E-01 | FALSE | 8.07E-01 | FALSE | 9.75E-01 | FALSE |
